# Microtubule modification defects underlie cilium degeneration in cell models of retinitis pigmentosa associated with pre-mRNA splicing factor mutations

**DOI:** 10.3389/fgene.2022.1009430

**Published:** 2022-09-13

**Authors:** Liliya Nazlamova, Suly Saray Villa Vasquez, Jenny Lord, Varshini Karthik, Man-Kim Cheung, Jörn Lakowski, Gabrielle Wheway

**Affiliations:** ^1^ Human Development and Health, Faculty of Medicine, University of Southampton, Southampton, United Kingdom; ^2^ Centre for Research in Biosciences, University of the West of England, Bristol, United Kingdom; ^3^ Clinical and Experimental Sciences, Faculty of Medicine, University of Southampton, Southampton, United Kingdom

**Keywords:** cilia, ciliopathies, retinitis pigmentosa, pre-mRNA splicing, photoreceptor

## Abstract

Retinitis pigmentosa (RP) is the most common cause of hereditary blindness, and may occur in isolation as a non-syndromic condition or alongside other features in a syndromic presentation. Biallelic or monoallelic mutations in one of eight genes encoding pre-mRNA splicing factors are associated with non-syndromic RP. The molecular mechanism of disease remains incompletely understood, limiting opportunities for targeted treatment. Here we use CRISPR and base edited *PRPF6* and *PRPF31* mutant cell lines, and publicly-available data from human *PRPF31* ^*+/−*^ patient derived retinal organoids and *PRPF31* siRNA-treated organotypic retinal cultures to confirm an enrichment of differential splicing of microtubule, centrosomal, cilium and DNA damage response pathway genes in these cells. We show that genes with microtubule/centrosome/centriole/cilium gene ontology terms are enriched for weak 3′ and 5′ splice sites, and that subtle defects in spliceosome activity predominantly affect efficiency of splicing of these exons. We suggest that the primary defect in *PRPF6* or *PRPF31* mutant cells is microtubule and centrosomal defects, leading to defects in cilium and mitotic spindle stability, with the latter leading to DNA damage, triggering differential splicing of DNA damage response genes to activate this pathway. Finally, we expand understanding of “splicing factor RP” by investigating the function of *TTLL3*, one of the most statistically differentially expressed genes in *PRPF6* and *PRPF31* mutant cells. We identify that *TTLL3* is the only tubulin glycylase expressed in the human retina, essential for monoglycylation of microtubules of the cilium, including the retinal photoreceptor cilium, to prevent cilium degeneration and retinal degeneration. Our preliminary data suggest that rescue of tubulin glycylation through overexpression of *TTLL3* is sufficient to rescue cilium number in *PRPF6* and *PRPF31* mutant cells, suggesting that this defect underlies the cellular defect and may represent a potential target for therapeutic intervention in this group of disorders.

## 1 Introduction

Retinitis pigmentosa (RP) (OMIM#268000) is the most common cause of hereditary blindness, affecting 1:2000 to 1:5000 people worldwide (Sharon and Banin, 2015; Verbakel et al., 2018). It is a progressive retinal dystrophy which is characterised by night blindness with restriction of peripheral vision developing into tunnel vision due to dysfunction of the rod photoreceptors of the retina. Later in the disease cones are also affected leading to loss of central and colour vision. Symptoms generally begin to develop in early adulthood, although age and rate of onset is variable, linked to some extent to the genetic lesion associated with the disease. It is inherited in a recessive, dominant and X-linked pattern, depending on the gene mutated in the affected individual.

RP occurs in isolation as a non-syndromic condition, or in combination with other clinical features as part of syndromes such as Usher’s syndrome (USH), Bardet-Biedl syndrome (BBS) and Joubert syndrome (JS). More than 70 genes are known to cause non-syndromic RP and around 200 genes have been associated with the syndromic and non-syndromic forms of the disease collectively. Around one third of genetic subtypes of RP are associated with defects in primary cilia, the cells’ signalling organelle (Estrada-Cuzcano et al., 2012; Bujakowska et al., 2017), and retinal dystrophies are a common feature of syndromic ciliopathies, diseases associated with defects in cilia. This is due to the presence of highly specialised ciliated cells in the retina; the photoreceptors. The outer segment of the rod and cone photoreceptor is considered to be a highly modified primary cilium (Oud et al., 2017). This structure is anchored at the base by a gamma tubulin basal body, from which extends the backbone of the cilium, the alpha- and beta-tubulin axoneme. Unlike the microtubules of the dynamic cytoskeleton, the axonemal microtubules are stable, and this stability is achieved through multiple post-translational modifications including acetylation, tyrosination, glutamylation and glycylation.

Non syndromic RP can also be caused by biallelic or monoallelic mutations in one of eight pre-mRNA splicing factors; *PRPF3* (Chakarova et al., 2002), *PRPF4* (Chen et al., 2014; Linder et al., 2014), *PRPF6* (Tanackovic et al., 2011), *PRPF8* (McKie et al., 2001), *PRPF31* (Vithana et al., 2001), *SNRNP200* (Zhao et al., 2009), *RP9* (*PAP1*) (Keen et al., 2002), *DHX38* (*PRPF16*) (Ajmal et al., 2014). Mutations in *PRPF8* and *PRPF31* each account for roughly 2% of cases of RP, *PRPF3* mutations account for around 1% of cases (Sullivan et al., 2006), and *SNRNP200* mutations are found in 1.6% of autosomal dominant RP patients (Bowne et al., 2013). *PRPF4*, *PRPF6*, *RP9* and *DHX38* mutations are rarer causes of RP. Collectively mutations in pre-mRNA splicing factor proteins are the second most common cause of autosomal dominant RP (Wheway et al., 2020). These proteins are all components of, or associated with, the U4/U6.U5 small nuclear ribonucleoprotein (tri-snRNP), a core component of the spliceosome, the huge molecular machine which catalyses splicing (Will and Luhrmann, 2011). Nearly all eukaryotic genes are spliced, and this process occurs in all cells which transcribe and express proteins. As a result it was a very unexpected discovery that mutations in these genes should cause a phenotype restricted to the retina, and for many years the molecular disease mechanism has remained poorly understood. Early work suggested that mutations in splicing factors cause spliceosome assembly or activity defects (Wilkie et al., 2006; Frio et al., 2008; Wilkie et al., 2008; Huranova et al., 2009; Yin et al., 2011), and higher demand for splicing, with concomitant higher expression of splicing factors, in the retina explains why only this tissue manifests disease in patients (Cao et al., 2011). However, studies of cells from human patients with PRPF mutations showed variable effects on splicing (Ivings et al., 2008; Wilkie et al., 2008). An alternative theory proposed was that transcripts of retinal-specific genes are more severely affected by splicing factor mutations than other groups of genes (Yuan et al., 2005; Mordes et al., 2007; Linder et al., 2011; Yin et al., 2011; Liu et al., 2015). This was supported by findings that different gene-specific splicing defects result from mutation of different core spliceosomal proteins (Pleiss et al., 2007; Papasaikas et al., 2015; Wickramasinghe et al., 2015). Another alternative model proposed that there are no splicing-related defects in this form of RP, and that the disease manifests due to the protein-folding response as mutant proteins aggregate in cells, which is more pronounced in retina due to UV-induced photooxidative damage (Comitato et al., 2007; Shinde et al., 2016). Oxidative stress has been shown in photoreceptor cultures derived from induced pluripotent stem cells (iPSCs) from RP patients with mutations in RP9 (Jin et al., 2011) and other work links the ischaemia-hypoxia response proteins to splicing factors (Schmidt-Kastner et al., 2008). Modelling retinal disease has advanced with the ability to culture retinal organoids from human patient-derived iPSCs. Prior to this the lack of clarity of disease mechanism in splicing factor RP was complicated by a lack of good mammalian models. Some Prpf knock-in and knock-out mice show no RP phenotype (Graziotto et al., 2008; Bujakowska et al., 2009; Graziotto et al., 2011), others show very late onset RPE defects, which could be confused with general age-related retinal defects (Graziotto et al., 2011; Farkas et al., 2014).

In 2015 a hypothesis-neutral high-throughput high-content siRNA knockdown screen designed to identify all proteins required for normal primary cilium structure and function revealed a major unexpected insight into pre-mRNA splicing factors and primary cilia. This work showed that a significant number of proteins involved in splicing including pre-mRNA splicing factors PRPF6, 8 and 31, are required for normal cilium growth and function, and that when these proteins are lost, there are profound effects on ciliogenesis (Wheway et al., 2015). Immunofluorescence imaging showed that PRPF6, PRPF8 and PRPF31 localise to the base of the cilium in mouse- and human-derived ciliated cell lines, consistent with earlier findings that a range of splicing factors, including PRPF8, interact with 32 known centrosomal proteins in a large-scale centrosomal proteomics study (Jakobsen et al., 2011). Immunoelectron microscopy showed that PRPF6 and PRPF8 localize to the apical inner segment, basal body complex, apical connecting cilium of photoreceptor cells and postsynapse of secondary retinal neurons. Fibroblasts from human patients with frame-shift deletion mutation in *PRPF31* exhibited cilia defects, with shorter and/or fewer cilia than controls. A *C. elegans prpf8* null mutant had a partial ciliogenesis defect, with truncated or fewer microtubules in the amphid channel sensory cilia. Collectively, these data suggest that these forms of RP associated with PRPF6, 8 and 31 mutations are retinal ciliopathies (Wheway et al., 2015). The observation of pre-mRNA splicing factor at the base of the cilium led some investigators to speculate whether these proteins have specific roles in RNA metabolism at this subcellular localisation, independent of their role in splicing, and that defects in these specific cilia functions underlie disease (Johnson and Malicki, 2019). This hypothesis has been strengthened by the recent findings that mRNAs are localised to the base of the cilium for localised translation of ciliary mRNAs into protein (Iaconis et al., 2017; Hao et al., 2021). Nucleo-cytoplasmic shuttling of an accessory splicing factor is required for development and cilia function, further suggesting that core pre-mRNA splicing factors may be involved in a similar process, moving between the nucleus and base of the cilium to control which mRNAs are expressed in the cilium in a highly time-dependent manner (Haward et al., 2021). In a further publication, protein interaction data showed that PRPF6, 8 and 31 interact with many proteins involved in processes beyond splicing, including translation, mRNA stability, mRNA export and DNA damage response (Boldt et al., 2016), further suggestion that these proteins have roles in addition to splicing and that it may be dysregulation of these functions which underlie RP. Indeed, PRPF31 has subsequently been shown to play a direct role in mitotic chromosome segregation, through binding to kinetochore and centromere components (Pellacani et al., 2018), which has impacts on mitotic progression and differentiation of retinal progenitor cells in zebrafish (Li et al., 2021). This stall in mitotic progression leads to accumulation of DNA damage in Prpf31 crispant zebrafish retinal progenitors, exacerbated by enrichment of mis-splicing of DNA damage repair pathway transcripts (particularly those with weak 5′ splice sites) in these mutants, leading to apoptosis (Li et al., 2021). Depletion of PRPF8 has similarly been shown to lead to defects in mitotic progression, with specific defects in splicing of transcripts involved in mitotic progression, especially those with weak 5′ splice sites (Wickramasinghe et al., 2015).

Further study in human iPSC-derived retinal organoids and retinal pigment epithelium (RPE) from patients with *PRPF31* mutations showed decreased efficiency of splicing in an E1A minigene reporter assay (Buskin et al., 2018). RPE from patient iPSCs also showed a substantial downregulation of SART1, a U5 snRNP protein important for the formation of the pre-catalytic spliceosomal B complex, but no changes in the expression of PRPF8 or PRPF4. Retinal organoids from patients showed differential expression of actin cytoskeleton, ciliary membrane, primary cilium, photoreceptor inner and outer segment, axon terminal and phototransduction proteins. Retinal organoids from patients with *PRPF31* mutations showed an enrichment of mis-spliced centriole and microtubule organisation genes, with skipped exons, retained introns, alternative 5′ and 3′ splice sites, and mutually exclusive exons. In both RPE and retinal organoids derived from *PRPF31* ^*−/−*^ patients, the most significantly mis-spliced genes were genes involved in pre-mRNA and alternative mRNA splicing via the spliceosome. This suggests that ciliogenesis, cilium function, and pre-mRNA splicing are all regulated by alternative splicing in the retina, and this is defective in patients carrying *PRPF31* mutations (Buskin et al., 2018).

To further resolve the function of pre-mRNA splicing factors in regulating ciliogenesis and cilium stability in the retina we generated clonal CRISPR and base edited cell lines for study of cilia, protein and splicing in these cell models of human disease.

## 2 Materials and methods

### 2.1 Cell culture

Wild-type and *PRPF31* ^*+/−*^ (heterozygous NC_000019.10:g.54123455_54123456insA (NM_015629.4:c.422_423insA) (p.Glu141fs)) (Nazlamova et al., 2020) hTERT-RPE1 cells (ATCC CRL-4000) were cultured in DMEM/F12 (50:50 mix) + 10% FCS at 37°C, 5% CO_2_, and split at a ratio of 1:8 once per week. Wild-type and *PRPF6* ^*+/c.2185C>T*^ (described below) HEK293 cells (ATCC CRL-1573) were cultured in high glucose DMEM) + 10% FCS at 37°C, 5% CO_2_, and split at a ratio of 1:8 once per week. Cells were routinely tested for mycoplasma infection using a PCR-based method.

### 2.2 Base editing

We removed cas9/GFP from PX461 (Addgene #48140) (Ran et al., 2013) and replaced with GFP/Zeocin from pTRACER-EF/V5-His A (Life Technologies). GFP/Zeocin expression was controlled by the CMV promoter on PX461.s gRNA GGC​GCG​GGA​AGC​CTA​TAA​CC (PAM AGG) to target *PRPF6* c.2185C > T p.Arg729Trp was cloned into the BbsI sites, for expression from the U6 promoter. This was co-transfected with pCMV-BE3 (Addgene #73021) (Komor et al., 2016) which contains the BE3 gene (Base Editor; cytidine deaminase) under the control of a CMV promoter, into HEK293 cells using PEI. Cells were selected with zeocin.

### 2.3 Analysis of on-target changes

A proportion of bulk edited cells were harvested for DNA extraction and PCR amplification of the relevant targeted region of *PRPF6* using OneTaq polymerase (NEB). PCR products were cleaned using ExoSAP-IT (Thermo Fisher) and Sanger sequencing was performed by Source Biosciences. Traces from base edited cell lines were analysed visually to identify C > T changes at the predicted mutation site. Base editing efficiency of *PRPF6* c.2185C > T in HEK293 cells was around 50%. Although we tried multiple times to replicate this in hTERT-RPE1 cells and 661W cells we were unsuccessful. We took the edited HEK293 cells forward for single cell isolation.

### 2.4 Single cell cloning

Cells were dissociated using Accutase at room temperature, counted and transferred to a conical tube. Cells were collected by centrifugation at 200 g and washed with sterile sort buffer (Ca & Mg free PBS, 25 mM HEPES pH 7.0, 1–2.5 mM EDTA and 0.5% BSA or 1%–2% FCS). Cells were collected again and resuspended at a concentration of 5–8×10^6^ cells/ml. Untransfected cells were used for gating cell size on the FACS Aria cell sorter (BD) and edited cells then sorted into 150 μL DMEM/F12 + 20% FCS + 10% antibiotic and antimycotic + 10 μM Y-27632 ROCK inhibitor (STEMCELL Technologies) into each well of a 96 well plate.

### 2.5 Cell fractionation

Cells were fractionated into nuclear and cytoplasmic fractions. Cells were collected by scraping into fractionation buffer (20 mM HEPES pH7.4, 10 mM KCl, 2 mM MgCl_2_, 1 mM EDTA, 1 mM EGTA) on ice, lysed through a 27 gauge needle, on ice. The nuclear pellet was collected by centrifugation at 720 × g, washed and dispersed through a 25 gauge needle. The supernatant containing cytoplasm was centrifuged at 10,000 g to remove mitochondria and any cell debris. The dispersed nuclear pellet was collected again by centrifugation at 720 × g, resuspended in TBS with 0.1% SDS and sonicated to shear genomic DNA and homogenize the lysate.

### 2.6 RNA extraction

RNA was extracted from fractionated samples using TRIzol Reagent (Thermo Fisher). RNA quality and concentration was measured using an RNA Nano chip on the Agilent Bioanalyser 2100. Samples with total RNA concentration ≥20 ng/μl, RIN ≥ 6.8 and OD 260/280 were taken forward for cDNA library preparation and sequencing.

### 2.7 cDNA library preparation and sequencing

cDNA libraries were prepared using Ribo-Zero Magnetic Kit for rRNA depletion and NEBNext Ultra Directional RNA Library Prep Kit library prep kit by Novogene Inc. Library quality was assessed using a broad range DNA chip on the Agilent Bioanalyser 2100. Library concentration was assessed using Qubit and q-PCR. Libraries were pooled, and paired-end 150 bp sequencing to a depth of 20 M reads per fraction (40M reads per sample) was performed on an Illumina HiSeq2500 by Novogene Inc.

### 2.8 Data processing

#### 2.8.1 Raw data quality control

Raw FASTQ reads were subjected to adapter trimming and quality filtering (reads containing N > 10%, reads where >50% of read has Qscore ≤ 5) by Novogene Inc.

Quality of sequence was assessed using FastQC v0.11.5 (https://www.bioinformatics.babraham.ac.uk/projects/fastqc/). No further data filtering or trimming was applied.

### 2.9 Data deposition

Raw FASTQ reads after adapter trimming and quality filtering (reads containing N > 10%, reads where > 50% of read has Qscore ≤ 5) were deposited on the Sequence Read Archive, SRA accession PRJNA622794.

### 2.10 Alignment to reference genome

Paired FASTQ files were aligned to GRCh38 human genome reference using GENCODE v29 gene annotations (Frankish et al., 2019) and STAR v2.6.0a splice aware aligner (Dobin et al., 2013), using ENCODE recommend options (3.2.2 in the STAR manual (https://github.com/alexdobin/STAR/blob/master/doc/STARmanual.pdf). The two-pass alignment method was used, with soft clipping activated.

### 2.11 Alignment quality control

BAM files sorted by chromosomal coordinates were assessed for saturation of known splice junctions were using RSeqQC v3.0.1 (Wang et al., 2012).

### 2.12 Differential gene expression analysis

Reads were counted using HTSeq (Anders et al., 2015) and differential gene expression analysis was performed with edgeR v3.24.1 (Robinson et al., 2010; McCarthy et al., 2012) with statistical significance expressed as a *p* value adjusted for a false discovery rate of 0.05 using Benjamini-Hochberg correction.

### 2.13 Alignment to reference transcriptome and transcript level abundance estimates

The tool Salmon was used to perform transcript abundance estimates from raw FASTQ files using selective alignment with a decoy-aware transcriptome built from GRCh38 (Patro et al., 2017).

### 2.14 Differential splicing analysis

rMATs v4.0.2 (rMATS turbo) (Shen et al., 2014) was used to statistically measure differences in splicing between replicates of wild-type and mutant sequence. BAM files aligned with STAR v2.6.0a two-pass method with soft clipping suppressed were used as input.

Pathway enrichment was carried out using Enrichr (Chen et al., 2013; Kuleshov et al., 2016).

### 2.15 Differental exon usage analysis

DEXSeq-Count v1.28.1.0 was used to count exons in sorted bam files, using GENCODE v29 gene annotations. The differential exon usage was then determined between the control sample and the PRPF31 siRNA samples, using the DEXSeq (R version 3.5.1), again using GENCODE v29 gene annotations.

### 2.16 Splice site strength measurement

Using splice site coordinates from STAR splice junction output files and rMATS output files, splice site sequences were extracted from GRCh38 using bedtools, and splice strength calculated using MaxEntScan (Yeo and Burge, 2004).

### 2.17 Protein extraction

Total protein was extracted from cells using 1% NP40 lysis buffer and scraping. Insoluble material was pelleted by centrifugation at 10,000 × g. Cell fractionation was carried out by scraping cells into fractionation buffer containing 1 mM DTT and passed through a syringe 10 times. Nuclei were pelleted at 720 × g for 5 min and separated from the cytoplasmic supernatant. Insoluble cytoplasmic material was pelleted using centrifugation at 10,000 × g for 5 min. Nuclei were washed, and lysed with 0.1% SDS and sonication. Insoluble nuclear material was pelleted using centrifugation at 10,000 × g for 5 min.

### 2.18 SDS-PAGE and western blotting

20 μg of total protein per sample with 2 × SDS loading buffer was loaded onto pre-cast 4%–12% Bis-Tris gels (Life Technologies) alongside Spectra Multicolor Broad range Protein ladder (Thermo Fisher). Samples were separated by electrophoresis. Protein was transferred to PVDF membrane. Membranes were incubated with blocking solution (5% (w/v) non-fat milk/PBS), and incubated with primary antibody overnight at 4°C. After washing, membranes were incubated with secondary antibody for 1 h at room temperature and exposed using 680 nm and/or 780 nm laser (LiCor Odyssey, Ferrante, Giorgio et al.), or incubated with SuperSignal West Femto reagent (Pierce) and exposed using Chemiluminescence settings on ChemiDoc MP imaging system (Bio-Rad)

### 2.19 Primary antibodies for WB

Mouse anti ß actin clone AC-15 (Sigma-Aldrich A1978) 1:4000

Rabbit anti-PRPF6 primary antibody (Proteintech) 1:1000

Mouse anti-PCNA-HRP (BioRad MCA1558P) 1:1000

Mouse anti-monoglycylated tubulin (TAP952) (Sigma Aldrich MABS277) 1:100

### 2.20 Secondary antibodies for WB

Donkey anti mouse 680 (LiCor) 1:20,000

Donkey anti rabbit 800 (LiCor) (Ferrante et al., 2001) 1:20,000

Donkey anti mouse HRP (Dako) 1:10,000

Donkey anti rabbit HRP (Dako) 1:10,000

### 2.21 Immunocytochemistry of cells grown on cover slips for regular confocal imaging

HEK293s were plated on Poly-D-Lysine and laminin double-coated glass cover slips then serum starved for 6 days to induce ciliogenesis. hTERT RPE1 cells were grown on glass coverslips and serum starved for 48 h to induce ciliogenesis. Cells were rinsed in warm DPBS and fixed in ice-cold methanol at −20°C for 5 min. Cells were then immediately washed with PBS, and incubated with blocking solution (1% w/v non-fat milk powder/PBS) for 15 min at room temperature. Coverslips were inverted onto primary antibodies in blocking solution in a humidity chamber and incubated at 4°C overnight. After 3 washes with PBS, cells were incubated with secondary antibodies and DAPI for 1 h at room temperature in the dark. After 3 PBS washes and 1 dH2O wash, cells were mounted onto slides with Mowiol.

When staining with anti acetylated alpha tubulin, cells were incubated on ice for 15 min prior to fixation to reduce background staining of cell body microtubules.

### 2.22 Immunocytochemistry of cells grown on cover slips for STED confocal imaging

HEK293s were plated on Poly-D-Lysine and laminin double-coated high performance (high tolerance) #1.5 glass cover slips (Zeiss) then serum starved for 6 days to induce ciliogenesis. Cells were rinsed in warm DPBS and fixed in ice-cold methanol at −20°C for 5 min. Cells were then immediately washed with PBS, and incubated with blocking solution (1% w/v non-fat milk powder/PBS) for 15 min at room temperature. Coverslips were inverted onto primary antibodies at 5x higher concentration than the normal concentration optimsed for regular confocal imaging, in blocking solution in a humidity chamber and incubated at 4°C overnight. After 3 washes with PBS, cells were incubated with secondary antibodies at 10x higher concentration than the normal concentration optimsed for regular confocal imaging without DAPI for 1 h at room temperature in the dark. After 3 PBS washes and 1 dH2O wash, cells were mounted onto slides with Prolong Gold.

### 2.23 Manual confocal image capture and analysis

Confocal images were obtained at the Centre for Research in Biosciences Imaging Facility at UWE Bristol, using a HC PL APO 63x/1.40 oil objective CS2 lens on a Leica DMi8 inverted epifluorescence microscope, attached to a Leica SP8 AOBS laser scanning confocal microscope. Images were captured using LASX software and deconvolution of selected images was carried out using Hyvolution (Leica/Huygens). Images were assembled in Image J, Adobe Photoshop and Illustrator. Percentage of ciliated cells was measured by counting cilia and counting nuclei in 5 fields of view. 50 random cilia from 5 fields of view were measured manually using the scale bar as reference.

### 2.24 STED confocal image capture

STED images were obtained at Wolfson Bioimaging Facility at the University of Bristol Image using a 592 nm STED laser and 660 nm STED laser sequentially using a 100x APO oil immersion lens on a Leica DMi8 inverted epifluorescence microscope, attached to a Leica SP8 AOBS laser scanning confocal microscope with STED module. Images were captured using LASX software, and assembled in Image J, Adobe Photoshop and Illustrator.

### 2.25 Imaging plate setup

Cells were plated at a density of 1 × 10^5^ cells ml^−1^ into 100 μl complete media per well in 96 well optical bottom Perkin Elmer ViewPlates. The outer wells were filled with media without cells to reduce edge effects. Cells were cultured for 48 h before media was changed to serum-free media. Cells were fixed 24 h later.

### 2.26 UV treatment

Media was removed from each well and replaced with 10 μl DPBS to ensure the cells did not dry out, but not so much that the UV wasn’t able to penetrate. The plate lid was removed, mock-treated wells covered with foil, and a total dose of 25 J/m^2^ UV delivered to exposed wells in a UVP CL-1000 Ultraviolet crosslinker. Media was immediately replaced and plates returned to the incubator.

### 2.27 Nucleofection of PRPF31 and TTLL3 constructs

1 μg PRPF31 construct or TTLL3 construct was nucleofected into 200,000 hTERT-RPE1 cells using 20 μl P3 + supplement and programme EA104 in strip cuvettes on the Lonza Nucleofector 4D. 80 μl complete pre-warmed media was immediately added to each cuvette in the strip and cell suspension was returned to the incubator to recover for 15 min before adding 25 μl from each cuvette to 4 wells of a 96 well optical bottom Perkin Elmer ViewPlate containing 100 μl pre-warmed complete media per well.

### 2.28 Immunocytochemistry and imaging of 96 well plates

Wells were emptied by inversion of plates, and washed with warm Dulbecco’s PBS (Sigma). DPBS was removed by plate inversion and cells were fixed with ice cold methanol for 5 min at −80°C. Methanol was removed by plate inversion and cells were washed twice with PBS and non-specific antibody binding sites blocked with 1% non-fat milk powder/PBS (w/v) for 15 min at room temperature. Cells were incubated with primary antibodies in blocking solution for 1 h at room temperature and secondary antibodies + DAPI for 1 h at room temperature in the dark. Mowiol was added to wells, and plates stored until imaging. Imaging was carried out on a Perkin Elmer Opera LX with 20x and 60x water immersion lenses at Wolfson Bioimaging Centre, University of Bristol.

### 2.29 Automated image analysis

Image analysis was performed using custom scripts optimized on CellProfiler (Carpenter et al., 2006). Analysis included nuclear recognition and counting, cell recognition, exclusion of border objects and counting of whole cells, cilia recognition and counting, and quantification of the percentage of whole cells with a single cilium. Median and median absolute deviation of untransfected cells were used to calculate robust z scores (Zhang, 2007; Chung et al., 2008; Birmingham et al., 2009) of cell number and percentage of whole cells with a single cilium in transfected cells.

### 2.30 Retinal organoid culture

The Mastershef7 hESC line was differentiated into retinal organoids using a previously published protocol. Cells were seeded on LN521 in Nutristem hESC XF media (Biological Industries, #05-100-1A) and allowed to reach near confluence. Once confluent differentiation was initiated by culture in embryoid body media [EB media; DMEM/F12 (Gibco), Knockout serum replacement (Gibco), 1x MEM non-essential amino acids (Gibco), 1x Glutamax (Gibco)] for 72 h in the presence of WNT inhibitor 3 μM IWR1e (Merck). On day 4, the media was changed to Neural Induction Medium [NIM; DMEM/F12 (Gibco), 1x MEM non-essential amino acids (Gibco), N2 supplement (Gibco), Heparin (Sigma), 1x Glutamax (Gibco)] and feeding continued three times a week to stimulate forebrain development. On day 12 cultures were transitioned to Retinal Differentiation Medium (RDM; DMEM (Gibco), F12 (Gibco), B27 supplement (Gibco), 1x Glutamax (Gibco) and 10% Fetal Bovine Serum (LifeTech) and cultures fed on alternating days. After 18 days of differentiation, RDM was supplemented with Taurine (100 μM, Sigma), T3 (20 nM, Sigma), IGF-1 (5 ng/ml, Sigma). Cultures were supplemented with Retinoic Acid (0.5 μM, Sigma) after 100 days. After 3 weeks, Optic Vesicles (OVs) begin to form which appeared as rounded neuroepithelial structures growing adjacent to a patch of cells that form retinal pigment epithelium (RPE). The OVs were manually excised between day 25–35 and cultured in RDM in low attachment 10 cm plates until analysis.

### 2.31 Retinal organoid immunocytochemistry and imaging

Samples comprised of 10–20 OVs were fixed in 4% PFA/PBS for 10 min at room temperature and subsequently washed three times in PBS for 10 min each. The OVs were then cryopreserved by soaking in a PBS/30% sucrose solution overnight at 4°C. Embedding was carried out using OCT compound and frozen samples stored at −80°C. Frozen blocks were sliced into 18 μm cryosections and transferred on glass slides. OCT compound was removed by a 15 min incubation in PBS at 37°C. Sections were then blocked for 1 h (PBS/0.1% Triton-x, 10% FBS, 1% BSA) and then incubated with the primary antibodies overnight at 4°C. The next day, primary antibodies were washed off and the samples were incubated with secondary antibodies for 1 h at room temperature. Slides were then mounted and sealed with nail polish. Images were produced, from independent samples with a Leica SP8 Confocal microscope and processed using ImageJ software.

### 2.32 Primary antibodies for immunocytochemistry

Rabbit anti-PRPF6 (Proteintech 23929-1-AP) 1:100

Mouse anti-PRPF6 (Santa Cruz sc-166889) 1:100/1:50 for STED imaging

Rabbit anti-ARL13B (Proteintech 17711-1-AP) 1:200

Rabbit anti-gamma tubulin (Abcam ab11317) 1:50 (for STED imaging)

Mouse anti-polyglutamylated tubulin (GT335) (Adipogen Life Sciences AG-20B-0020) 1:1000

Rabbit anti-XPA (Abcam ab85914) 1:200

Mouse anti-monoglycylated tubulin (TAP952) (Sigma Aldrich MABS277) 1:100

Rabbit anti-recoverin (Millipore AB5585) 1:1000

Mouse anti-CRX (M02) clone 4G11 (Sigma H00001406-M02)

### 2.33 Secondary antibodies for immunocytochemistry

Donkey anti mouse IgG AlexaFluor 488 (ThermoFisher) 1:500

Donkey anti rabbit IgG AlexaFluor 568 (ThermoFisher) 1:500

Goat anti rabbit IgG AlexaFluor 488 (ThermoFisher) 1:1000/1:100 for STED imaging

Goat anti mouse IgG AlexaFluor 568 (ThermoFisher) 1:1000

Goat anti mouse IgG Highly cross-absorbed AlexaFluor 555 (ThermoFisher) 1:100 (for STED imaging)

### 2.34 Statistical analysis

Data were tabulated, graphs were produced and statistical analyses were performed in GraphPad Prism v9.0.1 (GraphPad Software Inc., San Diego, CA, United States) unless otherwise indicated. Normality of data was assessed using Shapiro-Wilk test to inform whether to apply parametric or non-parametric statistical tests. Statistical analyses of single comparisons of two groups utilized Student’s t-test or Mann-Whitney U test for parametric and non-parametric data respectively. Results were considered significant if *p* ≤ 0.05, where *p ≤ 0.05, **p ≤ 0.005, ***p ≤ 0.001, ****p ≤ 0.0001.

## 3 Results

**FIGURE 1:**
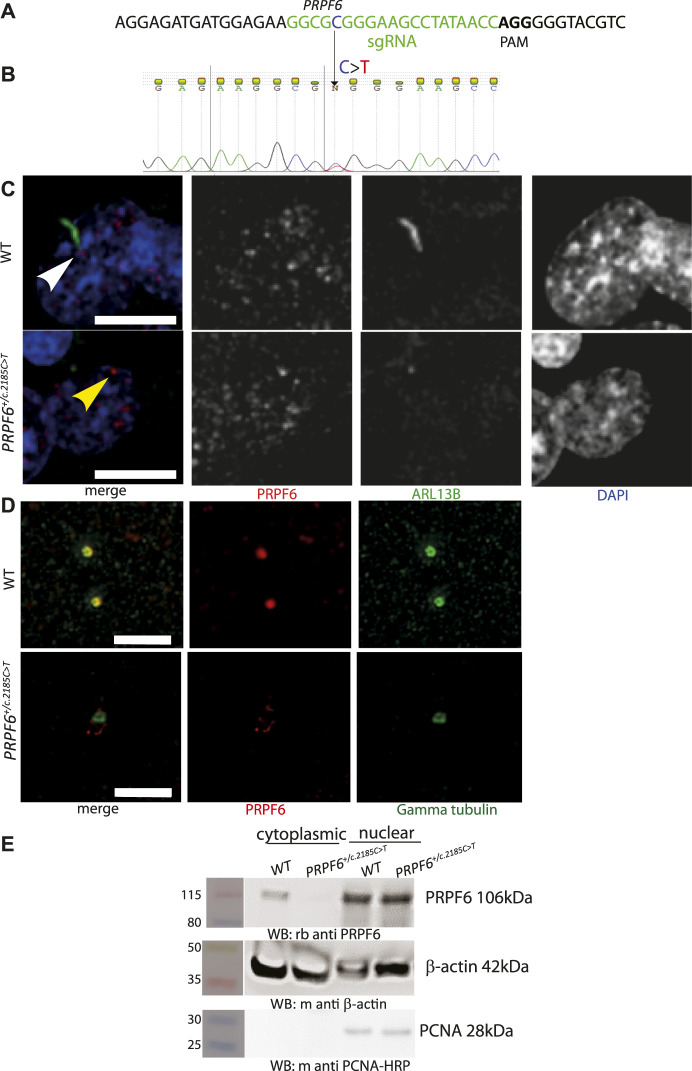
Generation and characterisation of *PRPF6* ^*+/c.2185C>T*^ base edited HEK293 cells. **(A)** Illustration of location and sequence of *PRPF6* guide RNA in green, with PAM sequencing in bold. **(B)** Electropherogram of PRPF6 sequence from of *PRPF6* ^*+/c.2185C>T*^ base edited HEK293 cells to confirm the heterozygous change. **(C)** Confocal microscopy images of WT and *PRPF6* ^*+/c.2185C>T*^ base edited HEK293 cells immunostained with anti PRPF6 (red) and ARL13B (green) antibodies, showing the ARL13B-stained cilium in wild-type (WT) cells (white arrowhead), lost in *PRPF6* ^*+/c.2185C>T*^ cells, and the loss of PRPF6 from the base of the cilium (yellow arrowhead). Scale bar 10 μm. **(D)** Stimulated emission depletion (STED) microscopy images of wild-type (WT) and *PRPF6* ^*+/c.2185C>T*^ base edited HEK293 cells immunostained with anti PRPF6 (red) and gamma tubulin (green) antibodies, showing colocalization of PRPF6 with the gamma tubulin-stained basal body in WT cells and loss of this colocalisation in *PRPF6* ^*+/c.2185C>T*^ cells. Scale bar 2 μm. **(E)** Western blot image showing presence of PRPF6 protein in the nucleus of WT and *PRPF6* ^*+/c.2185C>T*^ cells, but absence of PRPF6 protein from the cytoplasm of *PRPF6* ^*+/c.2185C>T*^ cells, beta actin is shown as a cytoplasmic loading control, and PCNA is shown as a nuclear loading control.

**FIGURE 2:**
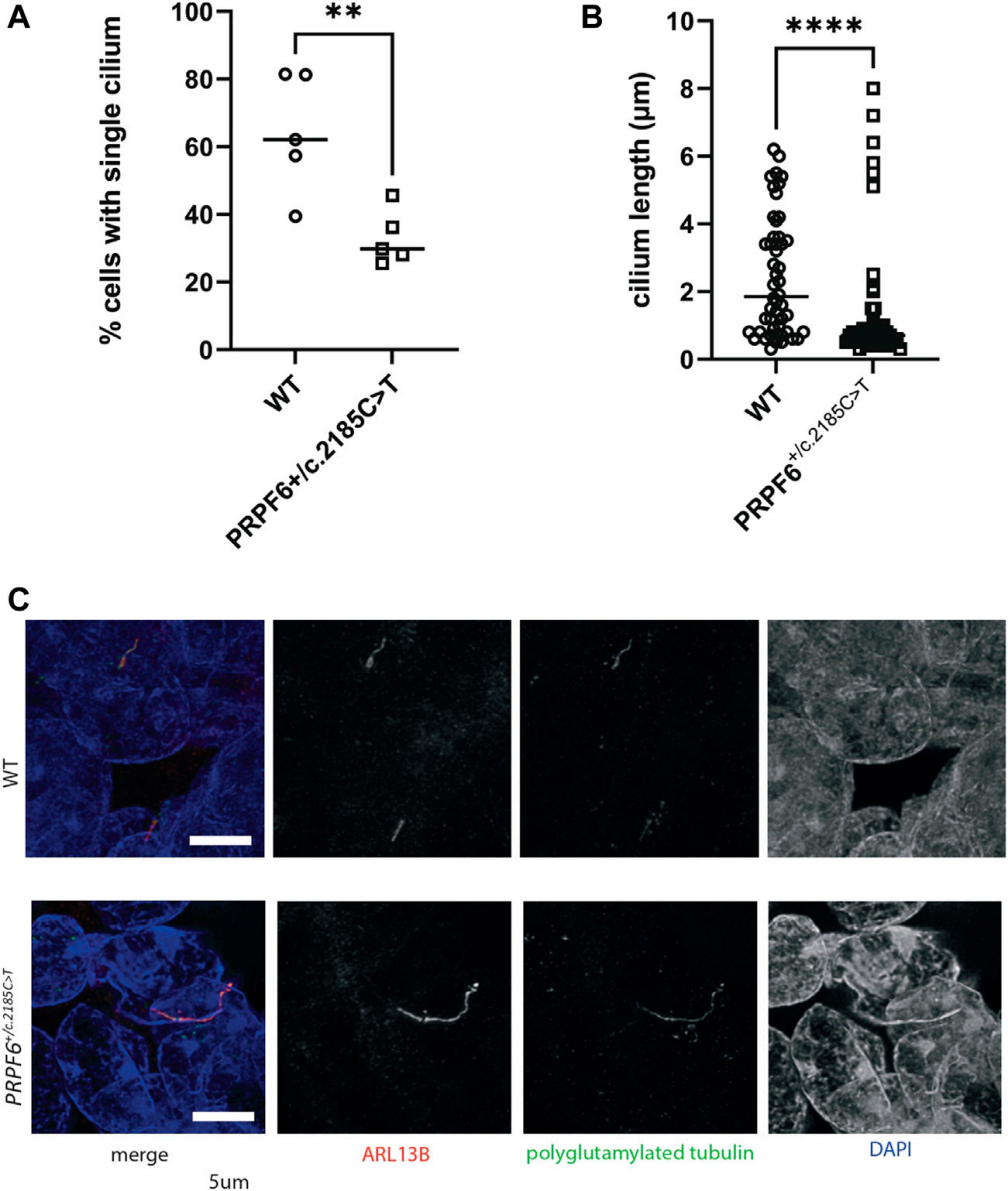
Quantification of ciliary phenotype in *PRPF6* ^*+/c.2185C>T*^ base edited HEK293 cells. **(A)** Dot plot of the percentage of cells with a single cilium in WT and *PRPF6* ^*+/c.2185C>T*^ cells, showing a statistically significant reduction in the percentage of cells with a single cilium in *PRPF6* ^*+/c.2185C>T*^ cells. Two-tailed unpaired *t* test, *p* value 0.0070, ** = *p* < 0.005, t, df t = 3.596, df = 8. One data point represents data from one cover slip (average from 6 fields of view per coverslip, 5 coverslips, each coverslip is one independent experiment). **(B)** Dot plot of single cilium length in WT and *PRPF6* ^*+/c.2185C>T*^ cells, showing a statistically significant reduction in cilium length in *PRPF6* ^*+/c.2185C>T*^ cells. Two-tailed Mann Whitney test, *p* value <0.0001 (exact), **** = *p* < 0.0001, Sum of ranks in column A, B 3095, 1955, Mann-Whitney U 680. One data point represents measurement of one cilium (measurements taken from individual cilia across 6 fields of view per coverslip, each coverslip is one independent experiment). **(C)** Confocal image of *PRPF6* ^*+/c.2185C>T*^ base edited HEK293 cells immunostained with anti ARL13B (red) and anti polyglutamylated tubulin (green) antibodies, showing the an example of a lengthened cilium in *PRPF6* ^*+/c.2185C>T*^ cells. Scale bar 5 μm.

Using third generation base editing (BE3) (Komor et al., 2016) vectors and a specific sgRNA (Figure 1A) we produced multiple clones of HEK293 cells with heterozygous knock-in point mutations in *PRPF6* [NC_000020.11:g.64027138C > T NM_012469.4(PRPF6):c.2185C > T (p.Arg729Trp)] previously reported in human autosomal dominant RP patients (Tanackovic et al., 2011), which we confirmed by Sanger sequencing (Figure 1B). We analysed the phenotype of 4 different mutant clones compared to 4 different wild-type clones which had undergone the same transfection and cloning process but not been edited. Immunofluorescence confocal imaging showed that, as has been shown in PRPF6 siRNA knockdown-treated cells (Wheway et al., 2015), *PRPF6* ^*+/c.2185C>T*^ mutant clones tended to lack cilia and PRPF6 staining could not be seen at the base of the cilium, whereas it could be seen in the wild-type sister clones (Figure 1C). PRPF6 staining in the nucleus appeared the same in mutants and wild-type cells (Figure 1C). To gain higher resolution images of the base of the cilium we immunostained cells with PRPF6 antibody and an antibody to gamma tubulin to label the basal body. This more clearly showed colocalization of PRPF6 with the basal body in the WT cells, and loss of this colocalization in the *PRPF6* ^*+/c.2185C>T*^ mutant clones (Figure 1D). Some faint PRPF6 staining could be seen to be loosely associated with the basal body in the mutant clones (Figure 1D) but when we fractionated cell lysates into cytoplasmic and nuclear fractions and analysed these by western blotting we could not detect PRPF6 in the cytoplasm in *PRPF6* ^*+/c.2185C>T*^ mutant clones (Figure 1E). We proceeded to quantify the cell defect seen in the *PRPF6* ^*+/c.2185C>T*^ mutant clones using deconvolution confocal imaging and manual counting of cilia and measurement of cilium length. This showed that *PRPF6* ^*+/c.2185C>T*^ mutant clones had statistically significantly lower percentage of cells with a single cilium (Figure 2A) and statistically significantly shorter cilia (Figure 2B), although with a small population of cells with long cilia (Figures 2B,C).

We extracted RNA from 3 independent *PRPF6* ^*+/c.2185C>T*^ mutant clones and 3 wild-type sister clones, prepared stranded cDNA libraries and performed paired-end RNA sequencing. We first carried out differential gene expression analysis, and defined a statistically significantly differentially expressed gene as one with a false discovery rate (FDR) adjusted *p*-value of <0.05 and a log_2_ fold change of greater than 1 or less than -1. This showed that 8 genes were statistically significantly differentially expressed in *PRPF6* ^*+/c.2185C>T*^ mutant clones compared to wild-type sister clones; 5 downregulated and 3 upregulated (Supplementary Table S1). Gene ontology (GO) term enrichment analysis using Enrichr (Chen et al., 2013; Kuleshov et al., 2016) showed statistically significant (adjusted *p*-value >0.05) enrichment of GO molecular function terms microtubule binding (GO:0008017; *p* = 0.003), tubulin binding (GO:0015631; *p* = 0.004), dynein complex binding (GO:0070840) and GO cellular component terms axon (GO:0030424; *p* = 0.001) and cytoskeleton (GO:0005856; 0.017) and GO biological process terms intermediate filament bundle assembly (GO:0045110; *p* = 2.098 × 10^−6^) and axon development (GO:0061564; *p* = 6.008 × 10^–4^).

**FIGURE 3:**
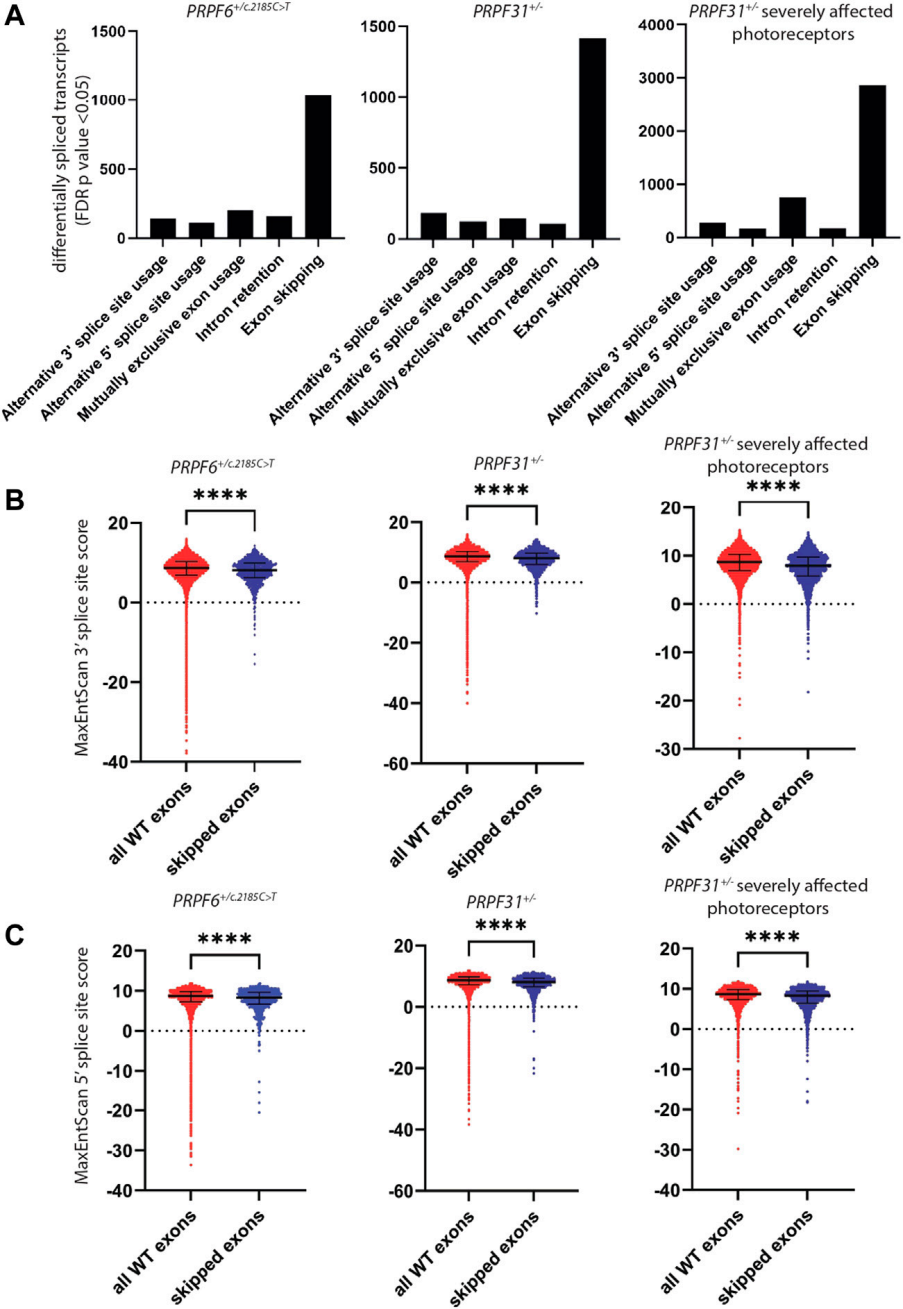
Analysis of differentially spliced transcripts and splice sites in *PRPF6* ^*+/c.2185C>T*^ cells, *PRPF31* ^*+/−*^ cells and retinal organoids derived from *PRPF31* ^*+/−*^ patients severely affected with RP. **(A)** Bar chart showing the number of cases of statistically significant (FDR *p* < 0.05) alternative 3′ splice site usage, alternative 5′ splice site usage, mutually exclusive exon usage, intron retention and exon skipping in *PRPF6* ^*+/c.2185C>T*^ cells, *PRPF31* ^*+/−*^ cells and retinal organoids derived from *PRPF31* ^*+/−*^ patients severely affected with RP compared to relevant controls, showing the abundance of exon skipping in all 3 cell/organoid types. **(B)** Dot plots showing MaxEntScan 3′ splice site score for every wild-type exon and every skipped exon identified in **(A)**, showing that skipped exons have lower 3′ splice site scores, i.e., weaker 3′ splice sites are skipped in *PRPF6* and *PRPF31* mutant cells. Individual data points plus median and interquartile range are shown ****p < 0.0001 Mann-Whitney in all cases. PRPF6 3′ WT median 8.680, SE media 8.150 *n* = 1039, PRPF31 3′ WT median 8.670, SE media 8.110 *n* = 1419, PRPF31 retinal organoids 3′ WT median 8.680, SE media 7.900 *n* = 2866. **(C)** Dot plots showing MaxEntScan 5′ splice site score for every wild-type exon (red) and every skipped exon (blue) identified in **(A)**, showing that skipped exons have lower 5′ splice site scores, ie weaker 5′ splice sites are skipped in *PRPF6* and *PRPF31* mutant cells. Individual data points plus median and interquartile range are shown ****p < 0.0001 Mann-Whitney in all cases. PRPF6 5′ WT median 8.700, SE median 8.345 *n* = 2866, PRPF31 5′ WT median 8.680, SE median 8.100 *n* = 1419, PRPF31 retinal organoids 5′ WT median 8.690, SE median 8.310 *n* = 2866.

To investigate differential splicing in our RNAseq dataset we used rMATS programme (Shen et al., 2014) to perform differential splicing analysis in our 3 independent *PRPF6* ^*+/c.2185C>T*^ mutant clones and 3 wild-type sister clones. We supplemented this analysis with differential splicing analysis of RNAseq data from 3 independent mutant *PRPF31* ^*+/−*^ hTERT-RPE1 clones and 3 independent wild-type sister hTERT-RPE1 clones, which we previously described (Nazlamova et al., 2020), and publicly available RNAseq data from day 150 retinal organoids derived from induced pluripotent stem cells (iPSCs) from 7 *PRPF31* patients severely affected with RP, compared to 6 unaffected controls (Buskin et al., 2018). Analysis included differential alternative 3′ splice site usage (A3′SS), alternative 5′ splice site usage (A5′SS), retention of introns (RI) and skipping of exons (SE). We accepted any event with FDR *p* < 0.05 as a significantly different splicing event. In *PRPF6* and *PRPF31* mutant cell lines and organoids the predominant differential splicing event was exon skipping (Figure 3A). To further investigate exon skipping we used DEXSeq to study differential exon usage in publicly available RNAseq data from 3 human organotypic retinal cultures treated with *PRPF31* siRNA compared to 1 non-targeting siRNA control (PRJNA509001) (Azizzadeh Pormehr et al., 2019). In these samples rMATS could not be used as only 1 control sample was available and rMATS requires replicate data.

Pathway and ontology enrichment analysis using Enrichr (Chen et al., 2013; Kuleshov et al., 2016) of differentially spliced genes in *PRPF6* and *PRPF31* mutant cell lines and organoids and *PRPF31* siRNA-treated organotypic retinal cultures showed an enrichment of gene ontology cellular component terms “centrosome”, “microtubule organising center”, “centriole” (Table 1) and an enrichment of pathways related to primary cilium development and DNA damage response (Table 2) across all *PRPF6* and *PRPF31* cells or retina. This is consistent with previous reports that centrosome and cilium transcript are differentially spliced in *PRPF31* mutants (Buskin et al., 2018; Azizzadeh Pormehr et al., 2020; Li et al., 2021).

**TABLE 1:** FDR adjusted *p* values from gene ontology enrichment analysis of differentially spliced exons in PRPF6 base edited cells, PRPF31 edited cells, PRPF31^+/−^patient-derived retinal organoids and PRPF31 siRNA-treated retinal cultures. Statistically significantly enriched GO terms and their FDR-adjusted *p* values are highlighted in bold.

	*PRPF6* ^*+/c.2185C>T*^ cells	*PRPF31* ^*+/−*^ cells	*PRPF31* ^*+/−*^ retinal organoids	*PRPF31* siRNA retinal cultures
Centrosome (GO:0005813)	**0.0031675420683829124**	**0.013562462**	**2.06E-04**	**1.81E-12**
Microtubule organizing center (GO:0005815)	**0.0031675420683829124**	**0.013309689**	**7.72E-04**	**5.66E-13**
Microtubule cytoskeleton (GO:0015630)	**0.021273276446275965**	0.82041854	0.388079555	**5.12E-06**
Microtubule organizing center part (GO:0044450)	0.3085426153586124	**0.01370083**	**0.010723266**	**0.002760253**
Centriole (GO:0005814)	0.6358832467779791	**0.028962917**	**0.011200905**	**0.043848651**
Mitochondrion (GO:0005739)	0.4365736275258775	**0.014404009**	0.131502434	0.264241865
PML body (GO:0016605)	0.7420592042778611	**0.025598309**	0.603125898	**1.00E-03**
Cytoskeleton (GO:0005856)	0.3199658477886017	0.932445377	**0.002457416**	**4.41E-05**
Spindle (GO:0005819)	0.5088351587057507	0.82041854	**0.006151164**	**0.008996979**
Vesicle coat (GO:0030120)	0.42128440695526675	0.82041854	**0.010723266**	**0.031007285**
Golgi membrane (GO:0000139)	0.8513466009078202	0.82041854	**0.037578067**	**0.002760253**
Spindle pole (GO:0000922)	0.5088351587057507	0.82041854	**0.049142179**	**0.003143342**

**TABLE 2:** FDR-adjusted *p* values from Wikipathway pathway enrichment analysis of differentially spliced exons in PRPF6 base edited cells, PRPF31 edited cells, PRPF31^+/−^patient-derived retinal organoids and PRPF31 siRNA-treated retinal cultures. Statistically significantly enriched pathways and their FDR-adjusted *p* values are highlighted in bold.

	*PRPF6* ^*+/c.2185C>T*^ cells	*PRPF31* ^*+/−*^ cells	*PRPF31* ^*+/−*^ retinal organoids	*PRPF31* siRNA retinal cultures
Genes related to primary cilium development (based on CRISPR) WP4536	0.3367102392058503	**2.33E-04**	**0.018904**	**4.98E-07**
DNA IR-damage and cellular response via ATR WP4016	0.7281307063162956	**0.01784**	0.999995	**0.036337**
Glycosylation and related congenital defects. WP4521	0.4937868948330625	0.787006	**0.018904**	0.82024
Nucleotide Metabolism WP404	-	0.777887	**0.041867**	0.877967

Analysis of Sashimi plots showed that this differential exon usage included skipping of single and multiple constitutive exons, skipping of novel unannotated exons which are included in wild-type cells, and skipping of constitutive exons alongside differential 3′ and 5’ splice site usage (Supplementary Figure S1).

It has been reported that regulation of splicing by PRPF8 is determined by 5′ splice site strength (Wickramasinghe et al., 2015), and that exons which are differentially spliced in the developing retina of *prpf31* mutant zebrafish have weaker 5′ splice sites (Li et al., 2021). To investigate the strength of 3′ and 5′ splice sites of exons skipped we extracted sequences of all splice sites used in control cells (>5 uniquely mapped reads per sample) and the splice sites of the exons skipped in mutant samples, and calculated splice site scores using MaxEntScan (Yeo and Burge, 2004). This showed a small but highly statistically significant difference in both 3′ and 5′ splice site strength of skipped exons in mutant samples compared to controls (Figures 3B,C). This suggests that subtle defects in spliceosome activity due to mutations in *PRPF6* or *PRPF31* predominantly affect efficiency of splicing of exons with weak splice sites. To investigate this further we performed enrichment analysis of genes with skipped exons with the weakest splice site scores (MaxEntScan < 1). Although there were no statistically significant (FDR *p* > 0.05) enrichments of pathways or ontologies we did observe consistent enrichment of genes with cellular component gene ontology term microtubule/centrosome/centriole/cilium. This suggests that exons in particular genes encoding microtubule/centrosome/centriole/cilium proteins may be particularly weak and susceptible to mis-splicing in cells with defects in pre-mRNA splicing. We hypothesised that the primary defect in cells was defective microtubule and centrosomal defects, and that this leads to defects in cilium stability and mitotic spindle stability, and the latter leads to DNA damage, triggering a secondary differential splicing of DNA damage response proteins. It has been well documented that differential splicing of DNA damage proteins is a central cellular response to DNA damage (Paulsen et al., 2009; Dutertre et al., 2010; Adamson et al., 2012; Shkreta and Chabot, 2015; Tresini et al., 2015).

**FIGURE 4:**
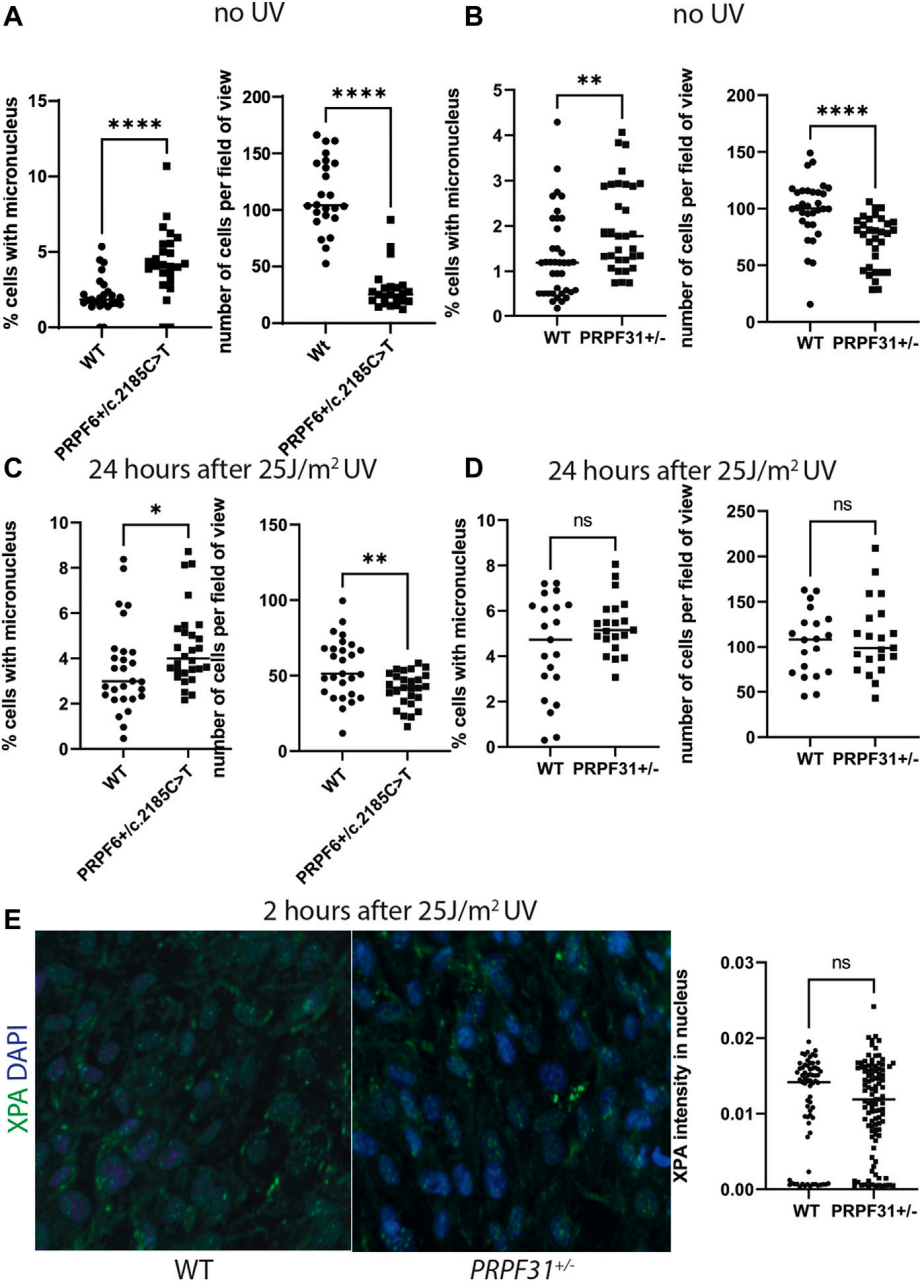
Analysis of micronucleus and cell number in *PRPF6* ^*+/c.2185C>T*^ cells and *PRPF31* ^*+/−*^ cells before and after UV exposure, and analysis of XPA nuclear staining in *PRPF31* ^*+/−*^ cells after UV exposure. **(A)** Dot plots showing percentage of cells with a micronucleus (left) and number of cells per field of view (right) in untreated WT cells and *PRPF6* ^*+/c.2185C>T*^ cells. *PRPF6* ^*+/c.2185C>T*^ cells have statistically significantly more micronuclei and statistically significantly fewer cells. **(B)** Dot plots showing percentage of cells with a micronucleus (left) and number of cells per field of view (right) in WT cells and *PRPF31* ^*+/−*^ cells. *PRPF31* ^*+/−*^ cells have statistically significantly more micronuclei and statistically significantly fewer cells. **(C)** Dot plots showing percentage of cells with a micronucleus (left) and number of cells per field of view (right) in WT cells and *PRPF6* ^*+/c.2185C>T*^ cells 24 h after 25 J/m^2^ UV. *PRPF6* ^*+/c.2185C>T*^ cells have statistically significantly more micronuclei and statistically significantly fewer cells, but these differences are less statistically significant than in untreated cells. **(D)** Dot plots showing percentage of cells with a micronucleus (left) and number of cells per field of view (right) in WT cells and *PRPF31* ^*+/−*^ cells 24 h after 25 J/m^2^ UV. *PRPF31* ^*+/−*^ cells do not have statistically significantly more or fewer micronuclei, nor statistically significantly more or fewer cells. **(E)** Confocal image (left) of WT and *PRPF31* ^*+/−*^ cells immunostained with XPA (green) and DAPI nuclear stain (blue) and dot plot (right) of XPA staining intensity in nucleus 2 h after 25 J/m^2^ UV exposure. No statistical difference is seen in mutants compared to WT cells.

To test this hypothesis we used our cell models to investigate DNA damage using high-content imaging. We assayed micronuclei number 24 h after treating cells with with 25J/m^2^ UV or giving a mock treatment. We found statistically significantly higher percentage of cells with micronuclei and lower cell number in mock treated *PRPF6* ^*+/c.2185C>T*^ mutants (Figure 4A) and *PRPF31* ^*+/−*^ cells (Figure 4B) than their respective wild-type sister clones, but the size and significance of this difference was reduced after UV exposure (Figures 4C,D). We also assayed intensity of XPA nuclear staining in *PRPF31* ^*+/−*^ cells and wild-type sister clones and found no statistical difference in mutants compared to wild type cells (Figure 4E). We conclude that mutant cells are more prone to genomic instability, but the DNA damage response is intact.

To further investigate the effect of differential splicing of microtubule regulating proteins on *PRPF6* and *PRPF31* mutant cells we focussed on one particular gene of interest, *TTLL3*, which was one of the most statistically differentially spliced genes in all mutants, and has some of the weakest splice sites of any gene identified. rMATS analysis identified multiple significant splicing defects in *TTLL3*, including intron retention, exon skipping and alternative 3′ and 5′ splice site usage. Most of these affected exons 5–7. The most significant intron retention in *PRPF6* ^*+/c.2185C>T*^ mutant cells was in *TTLL3*. Exon skipping in multiple transcripts of *TTLL3 was* highly significant in *PRPF6* ^*+/c.2185C>T*^ mutants. Exon skipping and A5′SS usage in *TTLL3* was also highly significant. *TTLL3* undergoes complex alternative splicing to produce 33 transcripts, including 18 protein coding transcripts which encode tubulin glycylase type 3, TTLL3. Glycylation is one of several post-translational modifications of tubulin in cilium axonemal tubulins which stabilise these structures. Whilst cell body microtubules undergo a range of post-translational modifications, glycylation is a modification unique to axonemal microtubules of both motile and primary cilia (Bosch Grau et al., 2013; Gadadhar et al., 2017). TTLL3 catalyses monoglycylation of the cilium microtubules and is required for ciliogenesis (Wloga et al., 2009; Rocha et al., 2014). Both mono- and polyglycylation coexist on axonemal MTs in most mammals, with three TTLL glycylases working together to generate polyglycylation of tubulins; TTLL3 and TTLL8 are initiating glycylases, TTLL10 elongates the polyglycylated chain (Rogowski et al., 2009). However, in humans polyglycylation is absent (Rogowski et al., 2009). GTEx expression data (GTEx Consortium, 2013; GTEx Consortium, 2015) suggests that TTLL3 is the only glycylase which is expressed in humans. *Ttll3* ^*−/−*^ mice lack glycylation in photoreceptors, which results in shortening of connecting cilia and slow retinal degeneration (Bosch Grau et al., 2017). Alterations in the balance of tubulin glycylation and glutamylation in photoreceptors has been shown to lead to retinal degeneration (Bosch Grau et al., 2017); absence of glycylation results in increased levels of tubulin glutamylation in photoreceptor cilia which leads to cilium degeneration. Furthermore, human mutations in *CCP5* (also known as *AGBL5*), a tubulin deglutamylase are associated with RP, presumably due to an increase in polyglutamylation (Kastner et al., 2015; Astuti et al., 2016; Branham et al., 2016; Patel et al., 2016). Another tubulin-modifying enzyme, *TTLL5*, a tubulin polyglutamylase, is also associated with cone-rod dystrophy type 9 (CORD9) and RP (Sergouniotis et al., 2014; Astuti et al., 2016; Bedoni et al., 2016) although it is thought that this is due to defective glutamylation of RPGR (Sun et al., 2016).

With the emerging role of enzymes involved in tubulin glycylation and glutamylation in retinal degeneration, the clinical and cellular phenotype associated with *PRPF6* and *PRPF31* mutations, TTLL3 is an extremely interesting candidate for further investigation.

**FIGURE 5:**
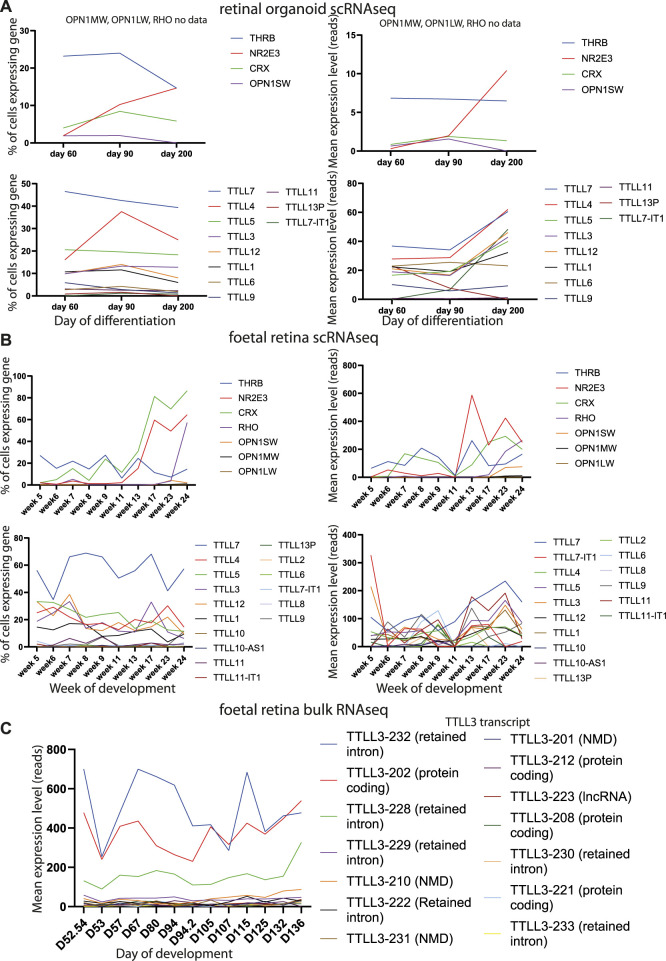
Analysis of retinal gene and TTLL gene expression in developing retinal organoids and foetal retina. **(A)** Line graphs plotting % of cells expressing genes (left) and mean expression level in raw reads of genes (right) in single cell expression datasets from retinal organoids. Data are shown from genes related to specific functions of different retinal cells (top) and TTLL genes (bottom) at different stages of retinal organoid differentiation. **(B)** Line graphs plotting % of cells expressing genes (left) and mean expression level in raw reads of genes (right) in single cell expression datasets from human foetal retinal. Data are shown from genes related to specific functions of different retinal cells (top) and TTLL genes (bottom) at different stages of retinal development. **(C)** Line graphs plotting mean expression level in raw reads of genes in bulk expression datasets from human foetal retinal. Data are shown for TTLL genes at different stages of retinal development.

**FIGURE 6:**
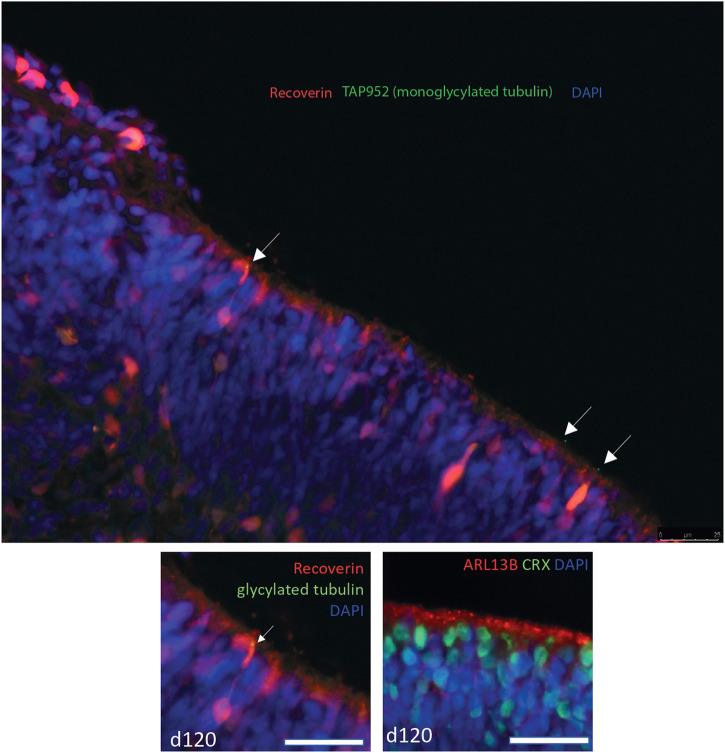
Confocal microscopy image of immunostaining of day 120 human embryonic stem cell derived retinal organoids. Recoverin is stained in red, monoglycylated tubulin is stained with TAP952 antibody in green, nuclei are stained with DAPI in blue. Monoglycylated tubulin staining is seen in recoverin positive cells in puncta reminiscent of basal bodies of connecting cilia (white arrows). The lower panel shows a magnified insert of the top panel (left) and a panel at the same magnification of the same D120 organoid stained with ARL13B (red) as a marker for cilia and CRX (green) photoreceptor cell marker.

We analysed publicly available RNAseq datasets from human retina to study expression of all tubulin tyrosine ligases (TTLLs) in different stages of human retinal development. Single cell sequence data from retinal organoids at day 60, 90 and 200 of differentiation (Collin et al., 2019) showed that *TTLL7* is the tubulin tyrosine ligase expressed by the largest proportion of cells across all time points (around half of all cells), with *TTLL4* and *TTLL5* also expressed by more than 20% of cells (Figure 5A). *TTLL3*, *12* and *1* are expressed by around 10% of cells, TTLL6, 9, 11 and 13P by a small number of cells and, consistent with GTex data, *TTLL8* and *TTLL10* not expressed at all (Figure 5A **)**. There is an increase in level of expression of most TTLL genes at day 200 (except *TTLL9*, *TTLL6*, *TTLL13P*) which correlates with development of mature outer segments at day 200 (Figure 5A). There was no obvious pattern of co-expression of any TTLL genes at any time point. Similar findings were observed in single cell RNAseq data from foetal human retina from week 5–week 24 (day 29–day 168) of development (Hu et al., 2019). The TTLL gene most commonly expressed in cells across all time points is *TTLL7* (expressed in around half of the cells of the retina) (Figure 5B). *TTLL4, 5, 3, 12* and *1* are expressed by one fifth to one third of cells of the retina (Figure 5B). *TTLL8* and *10* are not expressed at all. It is difficult to assess relative levels of expression across timepoints without normalisation but they would seem to be fairly consistent across TTLLs and timepoints, although expression of *TTLL7* and *TTLL3* was high in week 5, suggesting these genes may have a role in early retinal development (Figure 5B **)**. Transcript-level analysis of *TTLL3* in publicly available bulk RNAseq data from 13 whole human fetal retina samples spanning 12 time points (D52/54, D53, D57, D67, D80, D94 (2 samples), D105, D107, D115, D125, D132 and D136) (Hoshino et al., 2017), showed that 4 main protein coding transcripts of *TTLL3* (Ensembl transcript TTLL3-202, TTLL3-212, TTLL3-208 and TTLL3-221) are expressed across all timepoints of differentiation, with TTLL3-202 being the main protein coding transcript expressed. However, the highest level of expression of any transcript is of TTLL3-232, a retained intron transcript (Figure 5C). Together, the expression pattern of *TTLL3* and other TTLLs suggests that TTLL3 is the only tubulin glycylase expressed in the human retina, it is expressed by around 10%–25% of retinal cells throughout week 5 to week 24 of differentiation, and that the predominant expressed transcripts are 4834 bp TTLL-202 which encodes a 352 amino acid protein (the consensus coding sequence encodes a 915 amino acid protein) and a 5282bp transcript with a retained intron. RNAseq data does not give insight into when TTLL3 may play a functional role in tubulin monoglycylation in the retina, so we carried out immunostaining of monoglycylated tubulins in day 120 human embryonic stem cell derived retinal organoids; monoglycylation was seen specifically seen in RECOVERIN positive cells in D120 organoids in puncta reminiscent of basal bodies of connecting cilia (Figure 6).

**FIGURE 7:**
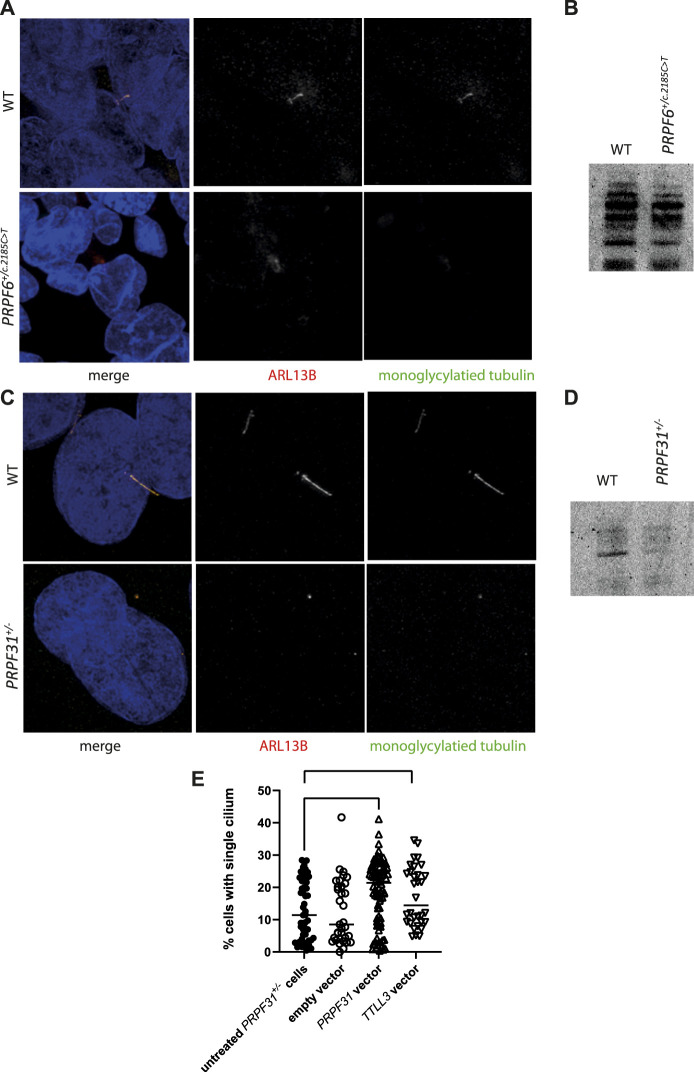
Analysis of monoglycylation in *PRPF6* ^*+/c.2185C>T*^ cells and *PRPF31* ^*+/−*^ cells, and rescue of ciliation in *PRPF31* ^*+/−*^ cells. **(A)** Confocal image of WT cells and *PRPF6* ^*+/c.2185C>T*^ cells immunostained with cilia marker anti ARL13B (red) and anti monoglycylated tubulin (green) antibody showing loss of monoglycylated tubulin from the cilium of mutant cells. **(B)** Western blot image showing reduction in levels of total monoglycylated tubulin in *PRPF6* ^*+/c.2185C>T*^ cells compared to WT sister clones. **(C)** Confocal image of WT cells and *PRPF31* ^*+/−*^ cells immunostained with cilia marker anti ARL13B (red) and anti monoglycylated tubulin (green) antibody showing loss of cilia from *PRPF31* ^*+/−*^ cells, but retention of monoglycylated tubulin at the basal body. **(D)** Western blot image showing reduction in levels of total monoglycylated tubulin in *PRPF31* ^*+/−*^ cells compared to WT sister clones. **(E)** Dot plot showing percentage of cells with a single cilium in untreated *PRPF31* ^*+/−*^ cells, and in *PRPF31* ^*+/−*^ transfected with an empty vector, a positive control rescue vector (*PRPF31*) or a *TTLL3* expression vector, showing statistically significant increase in percentage of cells with a single cilium in mutant cells transfected with either *PRPF31* or *TTLL3* construct.

With data suggesting that TTLL3 is required for monoglycylation in the retina photoreceptor cells, and evidence that differential splicing of this transcript is associated with *PRPF6* and *PRPF31* mutations and cilium defects, we investigated whether tubulin glycylation underlies cilium instability in PRPF6 and PRPF31 mutant cells. We immunostained cilia in our mutant cells and carried out western blotting with an antibody to monoglycylated tubulin. This showed lower levels of tubulin monoglycylation in *PRPF6* ^*+/c.2185C>T*^ and *PRPF31* ^*+/−*^ mutant cells compared to wild-type sister clones (Figures 7A–D). We next attempted rescue experiments in our *PRPF31* ^*+/−*^ mutant cells in which we transfected and expressed either an empty vector, a positive control rescue vector (*PRPF31*) or a *TTLL3* expression vector and assayed the effect on cilia using high content imaging and automated image analysis. This showed a rescue of cilium number in mutant cells transfected with either *PRPF31* and *TTLL3* (Figure 5C) suggesting that replacement of TTLL3 is sufficient to rescue cilium stability in PRPF31 mutant cells.

## 4 Discussion

It has been more than twenty years since mutations in pre-mRNA splicing factor genes were first identified as a cause of RP. Since then, considerable efforts have been made to understand why defects in proteins with constitutive functions in splicing in all cells only cause disease in the retina. In this study we used data from four complementary culture models; *PRPF6* and *PRPF31* mutant cell lines (Figures 1, 2), *PRPF31* patient-derived retinal organoids and *PRPF31* siRNA treated organotypic retinal cultures to corroborate a number of previous research findings, and provide new data to offer new insights into “splicing factor RP”. Consistent with previous studies, we show an enrichment of differentially spliced cilium and centrosomal genes in *PRPF6* and *PRPF31* mutant cells, organoids and organotypic cultures (Tables 1, 2; Supplementary Figure S1), and that these splicing differences are associated with weaker 5′ splice sites but for the first time we also show that these splicing changes are associated with weaker 3′ splice sites (Figure 3). No explanation for the specific mis-splicing of cilium and centrosomal genes in *PRPF6* and *PRPF31* mutant cells has ever been posited; our data provides a possible explanation, as it suggests that cilium and centrosomal genes are particularly enriched for weak splice sites, and are thus more prone to mis-splicing in cells carrying mutant pre-mRNA splicing factors. We hypothesise that this leads to cilium and centrosomal defects which are more pronounced in the retina due to the highly specialised structure and function of the retinal photoreceptor cilium. Our data is also consistent with previously published studies in identifying significant differential splicing of DNA damage response genes in *PRPF6* and *PRPF31* mutant cells and tissues (Figure 4, Tables 1, 2). Again, little explanation for this observation has been offered in the past. Our data suggests that, as is widely reported, differential splicing of DNA damage response genes is simply a marker of activation of the DNA damage response pathway in these cells, rather than symptomatic of a defect in the DNA damage response pathway. Our data suggests that the DNA damage response pathway is more highly activated in *PRPF6* and *PRPF31* mutant cells because of underlying microtubule and microtubule organising centre (centrosome) defects in these cells (Figure 4; Tables 1, 2). However, UV exposure is unlikely to play a role in RP disease progression *in vivo*, as little UV penetrates through the vitreous humor in the eye to reach the retina. In this study UV exposure is used as a research tool to investigate the effect of DNA damage on *PRPF6* and *PRPF31* mutant cells, but oxidative stress, from the high level of reactive oxygen species in the RPE cells, plays more of a role in retinal degeneration *in vivo* (Perdices et al., 2018; Murakami et al., 2020; Vingolo et al., 2022). It will be important to study the effects of reactive oxygen species on the rates of DNA damage response pathway in these model cell lines to further understand the likely disease mechanism of *PRPF6* and *PRPF31*-associated RP.

We further add to the existing literature by investigating the expression of one gene which is particularly significantly differentially spliced gene in *PRPF6* and *PRPF31* mutant cells; *TTLL3*, a tubulin glycylase. *TTLL3* contains a number of very weak splice sites, and undergoes complex splicing to produce a diverse array of transcripts, many of which were differentially expressed in *PRPF6* and *PRPF31* mutant cells. TTLL3 catalyses monoglycylation of the cilium microtubules and is required for ciliogenesis; alterations in the balance of tubulin glycylation and glutamylation in photoreceptors has been shown to lead to retinal degeneration; absence of glycylation results in increased levels of tubulin glutamylation in photoreceptor cilia which leads to cilium degeneration. *Ttll3* ^*−/−*^ mice develop retinal degeneration. Thus, the mis-splicing of *TTLL3* is highly interesting in the context of RP associated with *PRPF6* and *PRPF31* mutations. *TTLL3* RNA expression data in the human retina suggests that *TTLL3* is the only tubulin glycylase expressed in the human retina, expressed by around 10%–25% of retinal cells from week 5 to week 24 of differentiation (Figure 5). We also note that this timing of *TTLL3* RNA expression correlates with when we observe monoglycylated tubulins in RECOVERIN positive cells in human embryonic stem cell derived retinal organoids (day 120), further suggesting that this *TTLL3* RNA is translated into protein and plays a role in tubulin monoglycylation in the retina (Figure 6). However, RNA expression does not always correlate with protein expression, and the absence of TTLL3 protein expression data, in the form of western blot or immunofluorescence, is a notable gap in our paper, in the *Ttll3* ^*−/−*^ mouse study (Bosch Grau et al., 2017) and in the literature in general. It will be important to follow up this paper with studies of TTLL3 protein expression, if specific antibodies are available. We observe a reduction in monoglycylation in *PRPF6* and *PRPF31* mutant cells accompanied by a general loss of cilia (Figures 7A–D), although *PRPF6* mutant cells do retain some long cilia which exhibit glutamylation (Figure 2C), consistent with previously published literature showing an inverse relationship between glycylation and glutamylation levels in the *Ttll3* ^*−/−*^ mouse and *pcd* mouse (Bosch Grau et al., 2017). Our experiments with expression of exogenous *TTLL3* from a plasmid expression construct further suggest a functional role for *TTLL3* in ciliated retinal cells *in vitro* as this expression was able to rescue tubulin glycylation and ciliogenesis in PRPF mutant cells (Figure 7). This preliminary data demonstrating the rescue of tubulin glycylation and cilium number through exogenous *TTLL3* expression suggests that changes in *TTLL3* splicing and expression underlies the cilium defect in these cell models and may represent a potential target for therapeutic intervention in this group of disorders. It will be important to continue this work in human derived retinal organoids to investigate whether this is the disease mechanism *in vivo* and whether targeting of TTLL3 will be a useful therapeutic intervention for patients with this form of RP.

## Data Availability

The datasets presented in this study can be found in online repositories. The names of the repository/repositories and accession number(s) can be found below: https://www.ncbi.nlm.nih.gov/, PRJNA622794.

## Supplementary material

Supplementary Figure S1 Sashimi plots showing differential splicing of exons in genes with gene ontology terms “centrosome”, “microtubule organising center”, “centriole” and in pathways related to primary cilium development and DNA damage response in PRPF31^+/−^ cells and PRPF6^+/c.2185C>T^ cells.
